# Retraction: Antiproliferative and pro-apoptotic activity of melatonin analogues on melanoma and breast cancer cells

**DOI:** 10.18632/oncotarget.28800

**Published:** 2025-12-31

**Authors:** Giuliana Gatti, Valeria Lucini, Silvana Dugnani, Angela Calastretti, Gilberto Spadoni, Annalida Bedini, Silvia Rivara, Marco Mor, Gianfranco Canti, Francesco Scaglione, Annamaria Bevilacqua

**Affiliations:** ^1^Department of Medical Biotechnology and Translational Medicine, Università degli Studi di Milano, Milan, Italy; ^2^Department of Oncology and Hemato-oncology, Università degli Studi di Milano, Milan, Italy; ^3^Department of Biomolecular Sciences, Università degli Studi di Urbino “Carlo Bo”, Urbino, Italy; ^4^Department of Food and Drug, Università degli Studi di Parma, Parma, Italy; ^*^Authors contributed equally to this work

**This article has been retracted:** Oncotarget conducted an investigation into this paper after concerns were raised regarding Figure 5A, which contains three images of the same tumor, but described as a single photograph. The authors acknowledged this error as unintentional but agreed that it “affects profoundly the quality of the paper.” Additionally, we found several duplicated images of Western blots in Figures 7 and 8:

Figure 7A: The Akt image in DMSO-treated DX3 cells duplicates the tubulin image from UCM1037-treated cells.

Figure 7A: The Bax image from DMSO-treated DX3 cells duplicates the Bax image from DMSO-treated MCF-7 cells in Figure 8A.

Figure 7B: Two tubulin images (WM-115 cells) overlap with tubulin images in Figure 8A (MCF-7 cells).

In light of these irregularities, the Editorial decision was made to retract this article. Authors G. Spadoni, A. Bedini, S. Rivara, M. Mor, V. Lucini, and S. Dugnani have agreed to the retraction, stating that “improper modification of experimental images is a problem for data reliability and can affect the credibility of the paper. For this reason the undersigned authors ask for a formal retraction of the article, to preserve the scientific reputation of themselves and of their institutions.”

Original article: Oncotarget. 2017; 8:68338–68353. 68338-68353. https://doi.org/10.18632/oncotarget.20124

